# Psychological distress and its correlates among dental students: a survey of 17 Colombian dental schools

**DOI:** 10.1186/1472-6920-13-91

**Published:** 2013-06-26

**Authors:** Kimon Divaris, Ana Cristina Mafla, Laura Villa-Torres, Marisol Sánchez-Molina, Clara Liliana Gallego-Gómez, Luis Fernando Vélez-Jaramillo, Julián Andrés Tamayo-Cardona, David Pérez-Cepeda, Martha Ligia Vergara-Mercado, Miguel Ángel Simancas-Pallares, Argy Polychronopoulou

**Affiliations:** 1 Department of Pediatric Dentistry, UNC School of Dentistry, University of North Carolina-Chapel Hill, Chapel Hill, Brauer 228, CB#7450, Chapel Hill NC 27599, USA; 2 Grupo de Investigaciones en Odontología (GIOD), Facultad de Odontología, Universidad Cooperativa de Colombia–Pasto, Calle 18 No. 47-150, Pasto, Nariño, Colombia; 3 Department of Health Behavior, Gillings School of Global Public Health, University of North Carolina-Chapel Hill, CB #7440, Chapel Hill, North Carolina, USA; 4 Facultad de Odontología, Universidad Metropolitana, Calle 76 No. 42-78, Barranquilla, Atlántico, Colombia; 5 Facultad de Odontología, Universidad Cooperativa de Colombia - Medellín, Carrera 47 No. 37 Sur 18, Medellín, Antioquia, Colombia; 6 Facultad de Odontología, Universidad CES, Calle 10 A No. 22-04, Medellín, Antioquia, Colombia; 7 Institución Universitaria Colegios de Colombia sede Cali, Transversal 25 No. 18-21, Cali, Valle del Cauca, Colombia; 8 Facultad de Odontología, Fundación Universitaria San Martín, Carrera 15ª No. 60-80, Bogotá, Colombia; 9 Facultad de Odontología, Universidad del Sinú – Montería, Campus Elías Bechara Zainúm, Calle 38 Carrera 1 W Barrio Juan XXIII. Bloque 5 Piso 2 PBX, Montería, Córdoba, Colombia; 10 Facultad de Odontología, Universidad del Sinú - Cartagena, Av. Pedro de Heredia, Sector Amberes, Cartagena de Indias, Colombia; 11 Department of Preventive and Community Dentistry, School of Dentistry, National and Kapodistrian University of Athens, Goudi 11527, Greece

**Keywords:** Dental students, Psychological distress, Mental health, Psychopathology, Curriculum, Socioeconomic differences

## Abstract

**Background:**

Links between the demanding nature of studies in the health sciences, students’ personality traits and psychological distress have been well-established. While considerable amount of work has been done in medicine, evidence from the dental education arena is sparse and data from Latin America are lacking. The authors conducted a large-scale investigation of psychological distress among dental students in Colombia and sought to determine its curriculum and student-level correlates.

**Methods:**

The Spanish version of the Derogatis’ Symptoms Checklist Revised (SCL-90-R) was administered to all students officially registered and attending classes or clinics in 17 dental schools in 4 geographic districts of Colombia between January and April 2012. Additional information was collected on participants’ socio-demographic information and first career choice, as well as school’s characteristics such as class size. The Global Severity Index (GSI) score, a measure of overall psychological distress, served as the primary analytical endpoint. Analyses relied on multilevel mixed-effects linear and log-binomial regression, accounting for study design and sample characteristics.

**Results:**

A total of 5700 dental students completed the survey, a response rate of 67%. Pronounced gradients were noted in the association between socio-economic status and psychological distress, with students in higher strata reporting fewer problems. After adjustment for all important covariates, there was an evident pattern of increasing psychological distress corresponding to the transition from the didactic, to the preclinical and clinical phases of training, with few differences between male and female students. Independent of other factors, reliance on own funds for education and having dentistry as the first career choice were associated with lower psychological distress.

**Conclusions:**

Levels of psychological distress correlated with students’ socio-economic and study-level characteristics. Above and beyond the influence of person-level factors, variations in levels of distress paralleled specific transitional stages of the 5-year dental curriculum, providing opportunities for targeted interventions.

## Background

A considerable body of literature has established the links between the demanding nature of studies in the health sciences, students’ personality characteristics and manifestations of psychological distress [1-6]. Several reports indicate that students in medicine, dentistry, nursing and pharmacy experience high levels of perceived stress and psychological disturbance during the course of their studies, often at levels that are detrimental [3,7-9]. Prolonged psychological distress has been linked to a wide range of negative outcomes such as reduced academic performance, unprofessional conduct, burnout, and may even predispose to mental and physical disability [10-12].

While a substantial evidence-base on students’ well-being exists in medicine, data in the arena of dental education are relatively sparse. Numerous studies suggest that dental students experience or report high levels of stress [13,14], often higher compared to their medical counterparts [6,7,15] yet investigations of psychological distress are uncommon. Although limited by small sample sizes, findings by Lloyd and Musser [16], Henning *et al*. [2] and Naidu *et al*. [17] showed that alarmingly high proportions (30-50%) of dental students may be in the clinical range of psychological disturbance. Other investigators from Europe and Africa reported similar findings with regard to dental undergraduates’ general health and psychological distress [18,19]. Noteworthy, a recent qualitative study in a Colombian dental school provided insights into critical factors that may be particularly stress and anxiety-inducing [20].

We embarked upon this investigation to add to the knowledge base of dental students’ well-being and psychological distress. The literature reviewed by Alzahem and colleagues [13] offers a theoretical framing and justification for the selection of student- and institution-level, as well as extra-curricular factors as potential correlates of dental students’ psychological distress. For example, financial difficulties and “overcrowding” at school [19], social class [21], gender and minority status [16], first career choice [22], have been previously reported to be associated with dental students’ psychological well-being. Nevertheless, most studies have been limited by small sample sizes and no investigation to our knowledge has examined simultaneously ‘distal’ (e.g. socio-economic status, financial issues, career choice) and ‘proximal’ (e.g. gender, year of study) in among a large sample of dental students. Moreover, findings on the association of dental students’ psychological distress with age, marital status and gender have not been consistent [13,18]. Therefore, to inform educators and researchers, but also sensitize and guide stakeholders in an area that this topic has received little attention, we conducted a large-scale investigation in 17 dental schools in Colombia. Our aims were to identify school and student-level correlates of psychological distress among dental undergraduates, including curriculum, career choice and socio-economic factors. Our hypothesis was that extra-curricular factors, above and beyond traditional person-level and demographic ones, are important determinants of students’ psychological disturbance while in dental school.

## Methods

During the 2011–12 academic year there were 33 Colombian dental schools, operating in 18 Universities and 4 geographic regions, with a total of 13,944 enrolled students. In Colombia the majority students enroll in dental school right after high school and admissions are based primarily upon competitive national exams and school-conducted interviews. Most schools are private and have tuition rates that vary, but are generally considered high. Dental studies are structured around a traditional, mostly lecture-based 5-year curriculum. The first two years are focused on didactic activities on biomedical and dental introductory courses. During the 2^nd^ but mainly the 3^rd^ year students undergo laboratory and pre-clinical training. Years 4 and 5 entail clinical training. Empirical observations indicate that only a small proportion of dental students complete their studies within the 10 semesters of the regular curriculum; most require 1–3 extra semesters to graduate, whereas considerable proportions drop out.

The investigators initially contacted 22 schools from 13 Universities to participate in the study, and 17 (77%) schools agreed to do so. Although the resulting sampling frame of 8530 students does not constitute a probability sample of Colombian dental students, it represents 61% of the country’s entire dental student population.

After obtaining ethical approval, investigators contacted and sought to enroll all students officially registered and attending classes or clinics in each school between January and April 2012. Of 8530 eligible individuals 5700 agreed to participate, an overall response rate of 67%. Response rates varied and ranged from approximately 40% in UNAL - Bogotá and 42% in UAM - Manizales to 100% in UCC - Pasto, CURN - Cartagena and UMET -Barranquilla (Table 1). These differences were not strongly influenced by school characteristics. Nevertheless, we noted a statistically non-significant trend of lower response rate with increasing class (and school) size: “small schools”: 78%, “moderate size schools”: 75%, “large schools”: 67%; P = 0.5”.

**Table 1:** Description of the study sample frame and response rates, by geographical area, city and school

		**Colombian Dental Students**				
	**Enrolled**	**Sampled**		**Responded**		**Response Rate**
	**N**	**N**	**% of enrolled**	**n**	**column %**	**% of sampled**
Total	13,944	8530	61	5700	100	67
**Geographical area**						
West	5597	2769	49	1697	30	61
Central-East	5473	3369	62	1921	34	57
Caribbean	2570	2168	84	1921	34	89
Orinoquia	304	224	74	161	3	72
**City** - **School**						
Pasto – UCC	375	375	100%	375	6	100
Bucaramanga - USTA	945	945		599	10	64
Cartagena - CURN	258	258		258	5	100
Cartagena - UNICARTAGENA	445	445		405	7	91
Santa Marta - UNIMAGDALENA	540	540		378	6	70
Monteria – UNISINU	163	163		150	3	92
Medellín - UCC	648	648		380	7	59
Medellín - CES	558	558		350	6	88
Cali - UNICOC	570	570		334	6	59
Bogotá - UNAL	548	548		222	4	41
Bogotá - UNICOC	500	500		287	5	57
Bogotá - UAN	250	250		183	3	73
Bogotá - FUSM	1126	1126		630	11	56
Barranquilla - UMET	505	505		505	9	100
Villavicencio - UCC	224	224		161	3	72
Cartagena – UNISINU	257	257		225	4	88
Manizales - UAM	618	618		258	5	42

### Study instruments and procedures

The survey instrument contained an array of five questionnaires that covered the following domains: socio-demography (6 items), first career choice (1 item), school and study-level information (6 items), psychological distress [Spanish version of the Symptoms Checklist 90-Revised (SCL-90-R) [23] – 90 items], perceived sources of stress (Spanish version of the modified Dental Environment Stress (DES) questionnaire [24,25] - 36 items), self-efficacy (Spanish version of the General Self-Efficacy Scale [26] – 10 items) and burnout (Spanish adaptation of the Maslach Burnout Inventory-Student Survey [27] – 15 items). The present manuscript contains results of analyses that were focused on symptoms of psychological distress (SCL-90-R); another report [28] presents findings regarding the perceived academic stressors (DES) among this sample of students.

Students were approached during scheduled class or seminar times. Investigators explained the purpose, anonymous nature and voluntary character of the study. The survey was administered in paper and pencil form which required approximately 25 minutes for completion.

### Measures and variables

The main analytical endpoint (dependent variable) of the present report was psychological distress, as measured by the SCL-90-R. The SCL-90-R was introduced by Derogatis [29], and represents an evolution of earlier instruments measuring mental health, the SCL-90 [30] and the Hopkins Symptom Checklist [31]. The SCL-90-R is essentially a psychiatric self-report inventory, containing 90 items pertaining to various symptoms of psychological distress, such as “trouble remembering things” and “feeling nervous when you are left alone”. Participants were instructed to indicate how much distress each item has caused during the “last 7 days including today” on a 5-point scale ranging from 0: not at all to 4: extremely.

The instrument has been used among diverse populations, and was shown to perform well in terms of reliability and internal consistency. The SCL-90-R was originally reported to be capturing nine symptom dimensions, namely somatization, obsessive-compulsive, interpersonal sensitivity, depression, anxiety, hostility, phobic anxiety, paranoid ideation, and psychoticism. However, this factorial structure was not subsequently confirmed [32]. Instead, the Global Severity Index (GSI), the mean score of all items, is considered to be the best representation of an overall psychological distress dimension. Other indices of distress that can be derived from the inventory are the Positive Symptom Distress Index (PSDI), representing the average score of items scoring above zero and the Positive Symptoms Total (PST), representing the number of items scoring above zero [29]. The PSDI can be interpreted as a symptoms “intensity” measure and the PST as a symptoms “extent” measure. For clinical and consultation purposes, normative scores and thresholds were initially suggested for adolescent and adult psychiatric patients and non-patients [23], however, classifications derived from these standard scores have been of limited utility across populations and settings [33,34]. In fact, because the structure and properties of the instrument tend to vary between populations, it has been recommended to empirically establish them in each new study sample [35]. Nevertheless, there is agreement regarding differences in reporting of psychological disturbance with the SCL-90-R between males and females, and therefore, as most previous investigations we obtained and reported sex-stratified estimates.

We collected additional information regarding participants’ age [measured in years and coded as a categorical variable (under 18, 18- < 21, 21- < 24, and 24 and over) for descriptive and as a continuous variable for analytical purposes]; sex; study year (1–5); sources of funding for studies (own sources only, own sources and loans, loans only); working while studying (yes/no) and socio-economic level (1–6, where 1 is the lowest and 6 is the highest socio-economic stratum in Colombia). We also obtained information about the students’ first career choice (dentistry vs. other). With regard to school-level variables, we recorded its private or public character and the average class size [coded arbitrarily as small (<30), moderate (30–60) and large (>60 students)].

### Analytical approach

For initial data presentation and exploration we used descriptive statistics [simple proportions, means, standard deviations (*SD*), medians and ranges], overall and stratified by sex. We used X^2^ tests and a p < 0.05 criterion to assess the distribution of covariates between male and female participants, and a *t test* for the ‘age’ variable. The use of a *t test* for non-normally distributed data was supported by the central limit theorem applied to a large sample [36].

### Factor analysis

We explored the factor structure of SCL-90-R in the context of our study using iterated principal factor analysis with varimax rotation [37]. We empirically determined the factor structure by inspecting the corresponding Scree plot [38] and the proportion of ‘variance explained’. The inspection of the scree plot has been shown to perform better than Kaiser’s factor-retention criterion of eigenvalue ≥ 1, particularly in analyses with large number of items, where it consistently overestimates the number of factors to be retained [39]. Although we only used the GSI for analytical purposes, we generated and present internal consistencies (Cronbach’s *alphas*), mean scores, SD and 95% confidence intervals (CI) for all other SCL-90-R derived indices to offer an opportunity for comparisons with other samples and studies that employ these other metrics.

### Modeling of the psychological disturbance index (SCL-90-R)

The sample’s clustered nature of observations (respondents were “nested” in dental schools and these “nested” within cities and geographic regions) dictated the use of analytical methods based on multilevel modeling [40]. To determine the impact of student- and school-level factors on psychological disturbance throughout the 5-year curriculum we used multilevel mixed-effects multivariate linear regression of the GSI. We accounted for the clustering of observations and study sample design by specifying three nested random-effect terms, one for each sample “level”, geographic region, University/city, and school, confirming their inclusion by a Likelihood Ratio (LR) “chunk” test [41] and a p-value criterion of <0.2. In all models we entered *a priori* terms for age, sex, socio-economic status and study year. Inclusion of additional covariates was based on forward selection and LR tests with a p-value criterion of <0.2. To allow for non-homogeneous effects of age, socio-economic status and first career choice between males and females and across study years, we included a five-way interaction term between these variables. We based our inference on crude and adjusted beta coefficients and corresponding p-values using a p <0.05 criterion, as well as inspection of predictive marginal effects [42].

### Modeling of psychological morbidity “high scorers”

To quantify the impact of student and school-level factors on the prevalence of high psychological morbidity we used a second series of multivariate models based on log-binomial regression. First, we generated normalized GSI (T) scores centered at 50 separately for males and females and, as in previous studies [29,43-45], used a T score ≥63 definition for a psychological morbidity “high scorer”. This cut-off score resulted in classifying 8% of students of both sexes as “high scorers”. Subsequently, we fitted two log-binomial regression models separately for male and female “high scorer” and obtained adjusted prevalence ratios (PR) and 95% CI. Our choice of log-binomial versus the more common logistic regression was based on the fact that odds ratios obtained from logistic regression tend to overestimate the true effect size when the outcome under study is common (>20%) and because prevalence ratios are more readily interpretable compared to odds ratios in cross-sectional studies [46,47]. We used Stata version 12.1 (StataCorp LP, College Station, TX, United States) for all data analyses.

### Ethical approval

The study was approved by the Health Sciences Ethics Committee of Universidad Cooperativa de Colombia- Pasto (No. CECS02-12).

## Results

The mean age of the 5700 dental students was 21 years, with approximately two-thirds being female and having dentistry as their first career choice (Table 2). Most did not work while in dental school and were in socio-economic strata 2–4 reflecting low-to-middle socio-economic status. The number of participants gradually decreased across study levels, ranging from over 1300 1^st^ and 2^nd^ years to 686 5^th^ year students.

**Table 2:** Demographic characteristics of the study sample, stratified by sex

	**All respondents**		**Females**		**Males**		
	**N**	**col. %**	**n**	**row %**	**n**	**row %**	p-value^*^
Total sample	5700	100	3961	69	1739	31	
**Study year**							0.306
1^st^	1369	24	959	70	410	30	
2^nd^	1308	23	918	70	390	30	
3^rd^	1190	21	796	67	394	33	
4^th^	1147	20	805	70	342	30	
5^th^	686	12	483	69	203	31	
**Studies funding sources**							0.649
Own sources only	3302	58	2301	70	1001	30	
Own sources and loans	1897	33	1321	70	576	30	
Loans only	501	9	339	68	162	32	
**Working while studying**							<0.0005
Yes	1035	18	635	61	400	39	
No	4665	82	3326	71	1339	29	
**1**^**st **^**career choice: Dentistry**							0.120
Yes	3700	65	2597	70	1103	30	
No	2000	35	1364	68	636	32	
**Socioeconomic level**							0.043
1	379	7	245	65	134	35	
2	1248	22	859	69	389	31	
3	2366	42	1658	70	708	30	
4	1099	19	752	68	347	32	
5	442	8	319	72	123	28	
6	166	3	128	77	38	23	
**Age** (categorical)							<0.0005
Under 18	673	12	494	73	179	27	
18- <21	2398	42	1722	72	676	28	
21- <24	1804	32	1240	69	564	31	
24 and older	825	14	505	61	320	39	
**Age** (continuous)	Mean (SD)	Median (range)	Mean (SD)	Median (range)	Mean (SD)	Median (range)	<0.0005
	20.7(3.2)	20(15–54)	20.5(3.0)	20(15–53)	21.1(3.6)	21(15–54)	

^*^ Chi-square test for categorical variables and t test for the continuous age variable.

SD = Standard Deviation.

### Psychological distress

**Figure 1:**
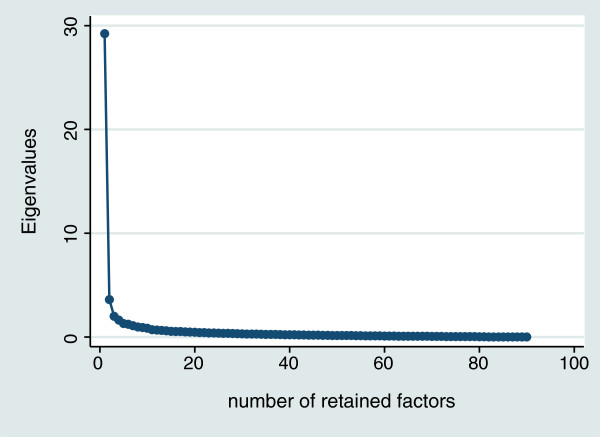
Scree plot of SCL-90-R exploratory factor analysis using iterated principal factor analysis with varimax rotation.

The exploratory iterated principal factor analysis confirmed the presence of one dominant factor in SCL-90-R, explaining over eight times the variance explained by the second factor (Figure 1). Cronbach’s *alpha* for the GSI was high, 0.98. The mean (SD) GSI scores were: overall—1.03 (0.69); females—1.08 (0.69); males—0.91 (0.68). Among female and male participants who had normalized T-scores of ≥63 and were classified as “high scorers” (8% of total), these estimates were 2.62 and 2.48, respectively. Although the GSI was used as our primary analytical endpoint, to enable comparisons with previous and future studies we present PST, PSDI and the 9 SCL-90-R dimensions scores in the supplemental material (Additional file 1: Table S1). To enable additional comparisons across studies and samples we additionally present the percentile distributions of the GSI, PST and PSDI indices by sex (Additional file 1: Table S2).

### Correlates of psychological distress

**Figure 2:**
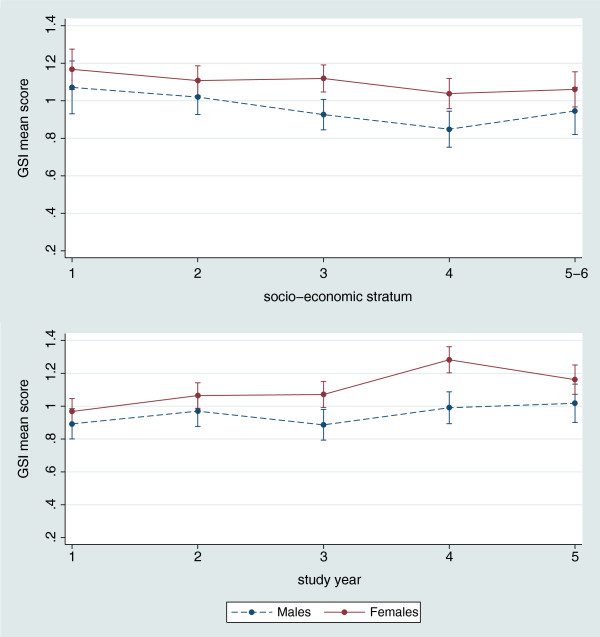
**Predictive margins of psychological disturbance (SCL-90-R Global Severity Index estimates) among male and female Colombian Dental students, across levels of socio-economic strata (top panel) and study year (bottom panel).** Predicted values and 95% confidence intervals are based on a multilevel mixed-effects linear regression model that included terms for age, sex, socio-economic status, marital status, financial support of dental studies, study level, first career choice, working while studying, class size and institution type.

The final multilevel regression model for GSI is presented in Table 3. After adjustment for all important covariates (age, marital status, working while studying, institution type and class size), sex, financial support sources, socio-economic status, career choice and study year, remained significantly associated with psychological distress. Females, students whose first career choice was not dentistry, those with loan-supported studies and in lower socio-economic strata reported higher levels of distress. The crude effect estimate corresponding to the difference between males and females was attenuated to a small degree (8%) after adjustment, with the multivariate beta coefficient indicating an adjusted difference of 0.16 points on the GSI scale. The strong effect of socio-economic status was virtually homogeneous among male and female participants (Figure 2, top panel). Above and beyond these associations, there was an evident pattern of increasing psychological distress corresponding to the transition from the didactic/basic science (1^st^ and 2^nd^), to the preclinical (3^rd^) and clinical (4^th^ and 5^th^ years) stage of the curriculum. Particularly among females, the transition into the clinical training phase was associated with a pronounced increase in GSI scores (Figure 2, bottom panel). Noteworthy, while the multilevel model’s random-effect term for school participation was significant (b = 0.13; 95% CI = 0.09, 0.19; P = 0.03), the terms for city/University and geographic reason were non-significant, indicating little additional variance explained after accounting for school participation.

**Table 3:** Results of multilevel* multivariate linear regression modeling of the SCL-90-R Global Severity Index (GSI) score

	**Beta coefficient**			
**Covariates**	**unadjusted**^†^	**adjusted**^‡^	**95% CI**	**p**-**value**
**Age** (continuous)	0.01	0.00	−0.01, 0.00	0.53
**Sex** (ref: male)				
Female	0.16	0.16	0.12, 0.20	<0.005
**Marital status** (ref: single)				
Married	0.08	0.05	−0.02, 0.13	0.16
**Working while studying**	−0.03	−0.01	−0.01, 0.04	0.73
**Financial support** (ref: own funds)				
Own funds + loans	0.08	0.05	0.01, 0.09	0.01
Loans only	0.14	0.11	0.05, 0.18	<0.005
**Dentistry was first career choice**	−0.11	−0.11	−0.15, -0.07	<0.005
**Socio**-**economic stratum** (ref: 1^st^)				
2^nd^	−0.05	−0.06	−0.13, 0.02	0.16
3^rd^	−0.07	−0.08	−0.16, 0.00	0.04
4^th^	−0.17	−0.16	−0.24, -0.07	<0.005
5^th^ or 6^th^	−0.12	−0.12	−0.21, -0.02	0.02
**Study year** (ref: 1^st^)				
2^nd^	0.09	0.09	0.03, 0.14	<0.005
3^rd^	0.07	0.08	0.02, 0.13	0.01
4^th^	0.25	0.25	0.19, 0.31	<0.005
5^th^	0.19	0.18	0.11, 0.25	<0.005
**Institution type** (ref: public)				
Private	−0.07	−0.08	−0.27, 0.11	0.39
**Mean class size per semester** (ref: <30)				
30-60 students	−0.03	−0.05	−0.23, 0.12	0.55
>60 students	−0.09	−0.05	−0.26, 0.16	0.66

^*^ Model included nested random effect terms to account for clustering of observations within school, city/University, and geographic region. †model included only random-effects for school, city/University and geographical area. ‡model included additionally all other variables presented in the table.

CI = Confidence Interval.

**Figure 3:**
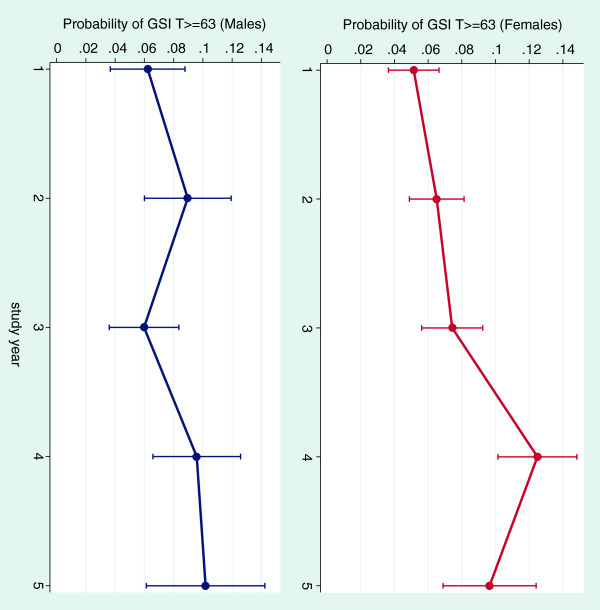
**Predictive margins of psychological morbidity “caseness” (SCL-90-R Global Severity Index T-score ≥63), across levels of study year for female and male dental students.** Predicted probabilities and 95% confidence intervals are based on a multivariate log-binomial regression model that included terms for age, sex, socio-economic status, marital status, financial support of dental studies, study level, first career choice, working while studying, class size and institution type.

In Table 4 we present results of the psychological disturbance analysis for males and females. Being married emerged as an important correlate of being a “high scorer”, with 83% increase in likelihood among males. Reliance on loans was strongly associated with distress particularly among females (PR = 2.01; 95% CI = 1.47, 2.76). Similarly, dentistry as a first career choice was important protective factor for female students, and was associated with 34% decreased prevalence (PR = 0.66; 95% CI = 0.64, 0.81). The clinical training stage was the peak period for “being a case” for both sexes vs. the first year (Figure 3): 4^th^ year among females—PR = 2.44 (95% CI = 1.70, 3.50) and 5^th^ year among males—PR = 1.54 (95% CI = 0.90, 2.94).

**Table 4:** Results of multivariate log-binomial regression modeling of high psychological disturbance (Global Severity Index T-score ≥63)

	**Females**			**Males**		
**Covariates**	**Prevalence Ratio**	**95% CI**	**p**-**value**	**Prevalence Ratio**	**95% CI**	**p**-**value**
**Age** (continuous)	0.99	0.95, 1.03	0.637	1.01	0.97, 1.06	0.571
**Marital status**						
Single	1.00	*referent*		1.00	*referent*	
Married	1.38	0.97, 1.96	0.073	1.83	1.07, 3.14	0.027
**Working while studying**	0.91	0.69, 0.18	0.471	0.85	0.59, 1.23	0.394
**Financial support**						
Own funds	1.00	*referent*		1.00	*referent*	
Own funds + loans	1.27	1.00, 1.61	0.052	1.49	1.05, 2.12	0.026
Loans only	2.01	1.47, 2.76	<0.0005	1.29	0.71, 2.26	0.420
**Dentistry was first career choice**	0.66	0.64, 0.81	<0.0005	0.83	0.60, 1.14	0.253
**Socio**-**economic stratum**						
1st	1.00	*referent*		1.00	*referent*	
2^nd^	1.08	0.67, 1.75	0.758	1.35	0.74, 2.47	0.329
3^rd^	1.17	0.74, 1.87	0.502	0.92	0.50, 1.71	0.793
4^th^	0.97	0.57, 1.63	0.900	0.88	0.43, 1.78	0.716
5^th^ or 6^th^	0.95	0.53, 1.70	0.850	0.85	0.36, 2.00	0.704
**Study year**						
1st	1.00	*referent*		1.00	*referent*	
2^nd^	1.27	0.87, 1.84	0.216	1.42	0.85, 2.38	0.184
3^rd^	1.44	0.98, 2.12	0.063	0.94	0.53, 1.68	0.842
4^th^	2.44	1.70, 3.50	<0.0005	1.54	0.91, 2.62	0.111
5^th^	1.89	1.23, 2.91	0.004	1.63	0.90, 2.94	0.104
**Institution type**						
Public	1.00	*referent*		1.00	*referent*	
Private	0.93	0.68, 1.26	0.627	0.72	0.48, 1.09	0.117
**Mean class size per semester**						
<30 students	1.00	*referent*		1.00	*referent*	
30-60 students	1.20	0.88, 1.61	0.245	0.94	0.60, 1.48	0.795
>60 students	1.22	0.88, 1.70	0.223	0.83	0.46, 1.49	0.524

## Discussion

In this study of student- and curriculum-level correlates of psychological distress among Colombian dental students we found that both demographic and socio-economic (sex, socio-economic status, and financial support sources) and study-related (career choice and study-year) characteristics influenced the levels of students’ psychological disturbance. This report builds upon a robust sample of almost 6 thousand students to add to the knowledge base of health science students’ psychological distress, and provides insights into this important issue among an under-studied group of dental students, in Latin America. Although direct comparisons with other samples and populations should be made with caution, the levels of psychological distress in this group of dental students were considerably higher compared to previously reported estimates among young non-clinical samples. The findings of possible associations between dental curriculum stage and psychological morbidity provide insights into educational events and transitions that may be sources of distress for dental undergraduates.

As mentioned previously, cross-cultural comparisons of SCL-90-R norms and scores is not well-supported [34]. Nevertheless, we empirically compared and found the present study’s GSI estimates (mean scores) to be higher compared to previous non-clinical sample norms among adolescents and students that have been published in the US—0.31 to 0.76 [23], New Zealand—0.88 [48], Spain—0.50 [49]. This observation is consistent with previous data indicating that dental students experience and demonstrate higher levels of stress and distress compared to age- and sex-matched norms, and compared to students of other disciplines [6,7,15-17]. Findings of high levels of psychological distress among dental students come as no surprise. One source of distress is likely inherent in the complex nature of dental education, which, beyond the acquisition of academic and interpersonal competencies, requires that undergraduates develop precision technical and surgical skills, and perform non-reversible operative procedures prior to graduation [14]. Additional sources may include students’ young age and personality traits [2,50], social, cultural and financial pressures unrelated to studies [51], and others.

As in previous investigations employing the same instrument (SCL-90-R), female dental students reported higher levels of psychological distress compared to their male counterparts. Given the ‘universality’ of this by-sex difference among normative and population-based samples, this finding should not be attributed to characteristics of the dental education environment. However, it is consistent with previous reports of females being more expressive of their emotions [52]. Findings relative to the protective or detrimental role of non-academic factors such as marital status, career choice, and financial pressures reiterate the necessity to view professional studies and students within their family, social and economic context. While factors such as financial difficulties and dissatisfaction with career choice [19,22] may exert additional pressures to students and be detrimental, other factors such as being married and working while studying may serve as buffers of coping and social support, and thus, be beneficial. Interestingly, in a survey of a US dental school, students rated non-academic support programs higher than ones that were focused on academic skills [53] providing support to the notion that non-academic influences are equally and perhaps more important than academic ones.

The variation of psychological distress across study years above and beyond all other demographic, socio-economic, and institution-level parameters is noteworthy. Distress levels increased overall, but pronounced spikes were evident in the transition to from the didactic/basic science, preclinical and clinical phases of the training. This is consistent with evidence of perceived stress escalating over time [52] or varying according to curricular landmarks such as transition from pre-clinical to clinical training [17,54]. These findings are likely demonstrations of excessive demands placed upon the students; work overload, little time, insufficient coping resources, non-academic distractions or combinations of those, can readily precipitate psychological disturbance and morbidity [54,55].

Our findings must be regarded in view of the study’s limitations. The participants did not represent a probability sample of all Colombian dental students, and response rates varied considerably between the 17 participating dental schools. Moreover, due to student attrition, the sample size decreased progressively from the 1^st^ to the 5^th^ study year. We cannot support that study non-participation was independent of psychological distress levels thus both these factors are potential threats to the external validity of our findings. Moreover, and although self-reported instruments are routinely used for screening purposes in psychiatry and social/behavioral epidemiology, students’ reports are also prone to biases of unknown magnitude and direction. Finally, our inferences with regard to differences in the levels of psychological distress by study-level were based on cross-sectional observations rather than longitudinal data, and therefore, should be interpreted with caution. A prospective survey design capturing student-level longitudinal data throughout the dental curriculum would enable investigators to make stronger inferences regarding the trends in psychological morbidity across the stages of dental training.

## Conclusions

The issue of alarmingly high levels of psychological distress during medical training is well-established, and evidence now accumulates in dentistry. Acknowledging the limitations of our cross-sectional survey, we support that both personal (sex, socio-economic status, and financial support sources) and curriculum or institution-level factors (career choice and study-year) are associated with dental undergraduates’ psychological distress. Under the conditions of our study, variations in levels of distress paralleled specific transitional stages of the 5-year dental curriculum, providing opportunities for targeted interventions in the curriculum. Based on these findings we further suggest that schools and states give special consideration to the students’ social and economic conditions, with the aim of allocating appropriate support to those most in need. Nonetheless, evidence of systematic application and evaluation of means to improve dental and health science students’ educational well-being is lacking and should be prioritized. It is imperative that such interventions, beyond evidence-based and effective, are participatory, socially and culturally-appropriate, and sustainable.

### Consent

Written informed consent was obtained from all participating dental students for the publication of this report, in compliance with the Health Sciences Ethics Committee of the Universidad Cooperative de Colombia - Pasto (ACT No. ECHS02-12).

## Abbreviations

CI: Confidence interval; GSI: Global severity index; PR: Prevalence ratio; PSDI: Positive symptom distress index; PST: Positive symptoms total; SCL-90-R: Symptoms checklist revised; SD: Standard deviation.

## Competing interests

The authors declare that they have no competing interests.

## Authors’ contributions

KD conducted the analysis and wrote the first draft of the manuscript; ACM conceived and coordinated study, participated in data collection and cleaning, and wrote the first outline of the manuscript; LVT participated in the study design, instrument piloting, and critically revised the manuscript; MSM, CLGG, LFVJ, JATC, DPC, MLVM, MASP participated in the planning of the study, data collection and entry, and critically revised the manuscript; AP oversaw the data design and analysis, and critically revised the manuscript. All authors approved the final version of the manuscript.

## Pre-publication history

The pre-publication history for this paper can be accessed here:

http://www.biomedcentral.com/1472-6920/13/91/prepub

## Supplementary Material

Additional file 1 Psychological distress estimates derived from the Symptom Check List 90-Revised (SCL-90-R). Click here for file
