# Supplementary material for: Psychological distress and its correlates among dental students: a survey of 17 Colombian dental schools
Source: BMC Med Educ. 2013 Jun 26;13:91. doi: 10.1186/1472-6920-13-91 (PMC3702479; doi:10.1186/1472-6920-13-91)
Supplement: Additional file 1 — Psychological distress estimates derived from the Symptom Check List 90-Revised (SCL-90-R). [file 1472-6920-13-91-S1.doc]

**Supplemental Table 1 Psychological distress estimates derived from the Symptom Check List 90-Revised (SCL-90-R)**

|  | | | | |
| --- | --- | --- | --- | --- |
|  |  | **All respondents** | **Females** | **Males** |
|  | Cronbach’s *alpha* | Mean (SD) | Mean (SD) | Mean (SD) |
| **Psychological Distress** |  |  |  |  |
| GSI | 0.98 | 1.03 (0.69) | 1.08 (0.69) | 0.91 (0.68) |
| PST |  | 45.2 (21.0) | 46.5 (20.2) | 42.1 (22.3) |
| PSDI |  | 1.90 (0.61) | 1.95 (0.62) | 1.80 (0.58) |
|  |  |  |  |  |
| **SCL-90-R proposed dimensions** |  |  |  |  |
| Somatization | 0.89 | 1.19 (0.86) | 1.29 (0.87) | 0.95 (0.78) |
| Obsessive-compulsive | 0.86 | 1.35 (0.86) | 1.39 (0.86) | 1.24 (0.84) |
| Interpersonal sensitivity | 0.84 | 0.85 (0.75) | 0.90 (0.76) | 0.74 (0.74) |
| Depression | 0.88 | 1.24 (0.84) | 1.34 (0.84) | 1.03 (0.79) |
| Anxiety | 0.86 | 0.98 (0.78) | 1.04 (0.79) | 0.86 (0.74) |
| Hostility | 0.83 | 0.99 (0.90) | 1.01 (0.90) | 0.94 (0.88) |
| Phobic anxiety | 0.79 | 0.56 (0.68) | 0.59 (0.69) | 0.49 (0.66) |
| Paranoid Ideation | 0.77 | 0.97 (0.81) | 0.98 (0.81) | 0.93 (0.80) |
| Psychoticism | 0.85 | 0.73 (0.73) | 0.73 (0.72) | 0.72 (0.75) |
| SD = Standard Deviation | | | | |

**Supplemental Table 2 Distribution of psychological distress scores derived from the Symptom Check List 90-Revised (SCL-90-R)**

|  | | | | | | | | |
| --- | --- | --- | --- | --- | --- | --- | --- | --- |
|  | **GSI** | |  | **PST** | |  | **PSDI** | |
|  | Females | Males |  | Females | Males |  | Females | Males |
| **Percentiles** |  |  |  |  |  |  |  |  |
| 5^th^ | 0.20 | 0.11 |  | 13 | 8 |  | 1.11 | 1.06 |
| 10^th^ | 0.30 | 0.20 |  | 20 | 13 |  | 1.21 | 1.13 |
| 20^th^ | 0.47 | 0.32 |  | 28 | 20 |  | 1.38 | 1.28 |
| 25^th^ | 0.54 | 0.40 |  | 32 | 24 |  | 1.47 | 1.36 |
| 33^rd^ | 0.67 | 0.49 |  | 36 | 30 |  | 1.58 | 1.45 |
| 50^th^ | 0.93 | 0.76 |  | 46 | 40 |  | 1.86 | 1.70 |
| 63^rd^ | 1.20 | 0.99 |  | 54 | 49 |  | 2.08 | 1.90 |
| 66^th^ | 1.27 | 1.04 |  | 56 | 52 |  | 2.15 | 1.95 |
| 75^th^ | 1.49 | 1.29 |  | 62 | 59 |  | 2.33 | 2.13 |
| 80^th^ | 1.62 | 1.46 |  | 65 | 64 |  | 2.45 | 2.24 |
| 90^th^ | 2.04 | 1.84 |  | 74 | 74 |  | 2.82 | 2.58 |
| 95^th^ | 2.41 | 2.27 |  | 80 | 81 |  | 3.12 | 2.95 |
| GSI = Global Severity Index  PST = Positive Symptoms Total  PSDI = Positive Symptom Distress Index | | | | | | | | |
